# Effect of Tunnel Drilling Portal on Femoral Tunnel Entry Aperture’s Location in Arthroscopic Anterior Cruciate Ligament Reconstruction

**DOI:** 10.7759/cureus.21948

**Published:** 2022-02-05

**Authors:** Ahmed Abdul Ghaffar, Rajesh Arora, Atul Agrawal, Arvind Kumar, Rajesh Maheshwari

**Affiliations:** 1 Orthopaedics, University College of Medical Science & Guru Teg Bahadur Hospital, New Delhi, IND; 2 Orthopaedics, Himalayan Institute of Medical Sciences, Dehradun, IND; 3 Orthopaedics, Hamdard Institute of Medical Sciences and Research, New Delhi, IND

**Keywords:** medial portal, femoral tunnel, far medial, arthroscopy, anterior cruciate ligament (acl)

## Abstract

Introduction

Transportal techniques for femoral tunnel drilling have the advantage of anatomical anterior cruciate ligament reconstruction, which was earlier difficult to achieve through transtibial femoral tunnels. However, the medial arthroscopic portal used for femoral tunnel drilling in single-bundle anterior cruciate ligament reconstruction (ACLR) has not been uniformly placed in different studies. Therefore, we performed a computed tomography-based analysis to compare the femoral tunnel entry aperture of the ACLR cases that used the standard AM portal and those using a far medial portal for femoral tunnel drilling.

Methods

We retrospectively reviewed computed tomography images of patients who underwent isolated single-bundle ACLR in our institute with either standard anteromedial portal or the far medial portal used for the femoral tunnel drilling. The femoral tunnel aperture's depth and height, measured using the quadrant method, were compared between the two portal methods.

Results

A total of forty-two case records were reviewed, sixteen belonging to standard anteromedial portal technique and twenty-six belonging to far medial portal technique. The tunnels created through the far AM portal were significantly shallower (more anterior) and inferior than the standard AM portal-created femoral tunnels.

Conclusion

The choice of drilling portals can influence transportal femoral tunnel drilling. A tendency towards anterior and inferior positioning of the femoral tunnel entry aperture has been observed when a far medial arthroscopic portal is used for femoral tunnel drilling. Therefore, care must be taken to ensure that the drilling guide pin position does not change when the reamer is passed over it.

## Introduction

Portal placement is crucial for successful anterior cruciate ligament (ACL) reconstruction. Transportal techniques for femoral tunnel drilling have the advantage of anatomical ACL reconstruction (ACLR), which was earlier difficult to achieve through transtibial femoral tunnels [1]. Conventionally, an anteromedial (AM) portal has been used for drilling the femoral tunnel [2]. The AM portal allows drill placement through the anatomical location of the femoral footprint of the ACL. While single bundle ACLR is the most preferred procedure, the transportal approach also helps double-bundle ACLR considering the direct access to the ACL femoral footprint and better control over drilling tracks [3]. However, the medial arthroscopic portal for single-bundle ACLR has not been uniformly placed in different studies [4-6]. Some authors have advocated for a medial portal close to the patellar tendon [2,7,8]. 

In contrast, others preferred more medialized portals, a medial accessory portal or a far medial portal [3-6,9]. The impact of portal medialization on the location of the femoral tunnel entry aperture has not been adequately addressed in the literature. The location of the entry aperture will ultimately impact the orientation of the ACL graft, and thus the clinical outcomes as well [10,11]. We performed computed tomography (CT) based analysis to compare the femoral tunnel entry aperture of the ACLR cases that used the standard AM portal and those using a far medial portal for femoral tunnel drilling.

## Materials and methods

We retrospectively reviewed clinical and radiological records of patients who underwent isolated single-bundle ACLR from Jan 2015 onwards. We included only those cases with postoperative CT digital images available and portal for femoral tunnel drilling specified in the operative notes as either standard AM portal or far medial portal. The standard AM portal was defined as a medial portal approximately 1 cm medial to the patellar tendon and just inferior to the inferior pole of the patella [2]. In the far AM portal technique, the optimal position for the accessory portal was located at a site that was as medial as possible but without injuring the medial femoral condyle by a reamer and as low as possible while avoiding the medial meniscus. We also noted the included cases' demographic characteristics (age, gender, BMI, and height). 

**Figure 1:**
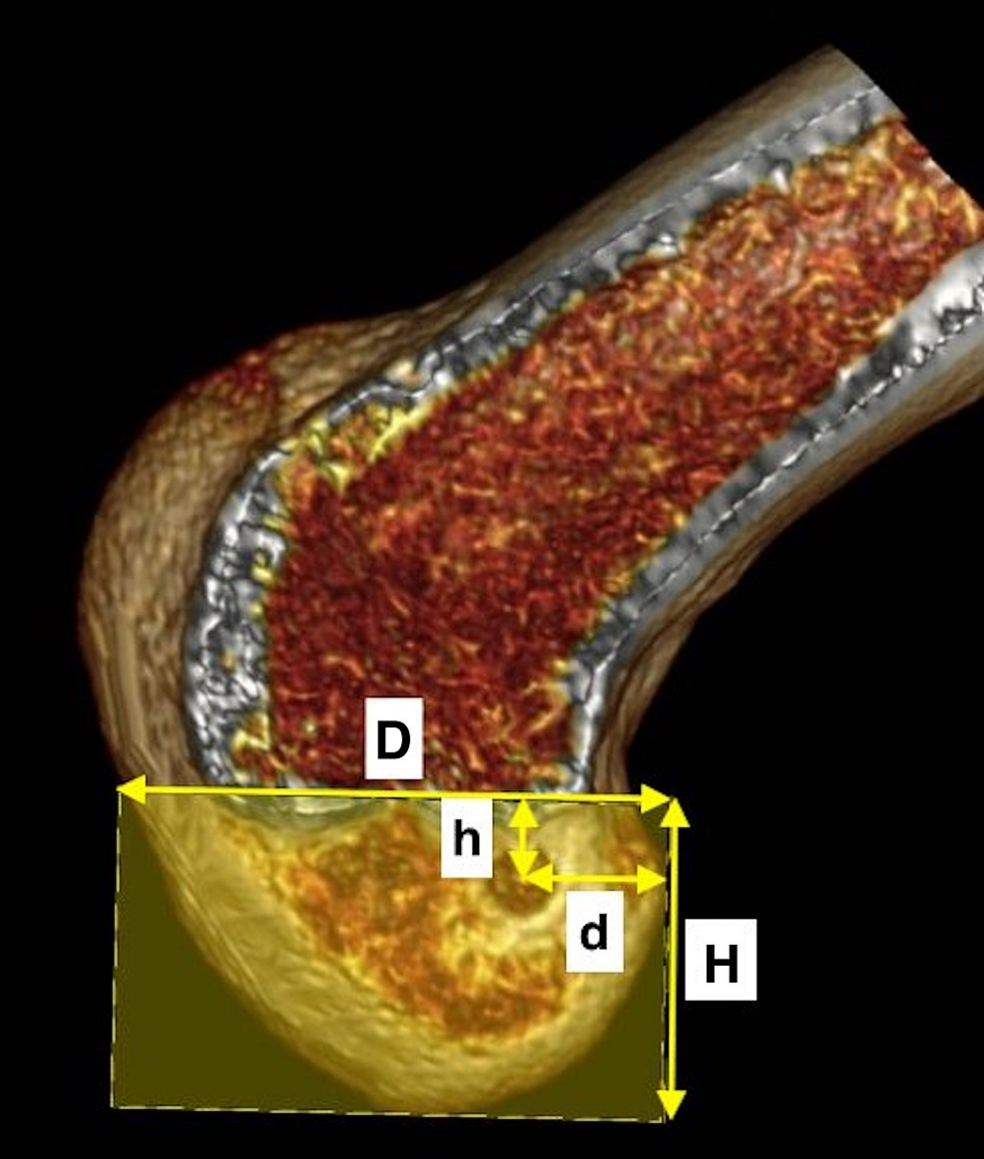
A representative three-dimensional computed tomography image of a distal femoral sagittal section through the Blumensaat line. The femoral tunnel entry aperture’s depth (d) is measured as a proportion of the rectangle’s overall anteroposterior extent (D) drawn using the quadrant method. Similarly, the femoral tunnel entry aperture’s height (h) is measured as a proportion of the rectangle’s overall superior-inferior extent (H).

**Figure 2:**
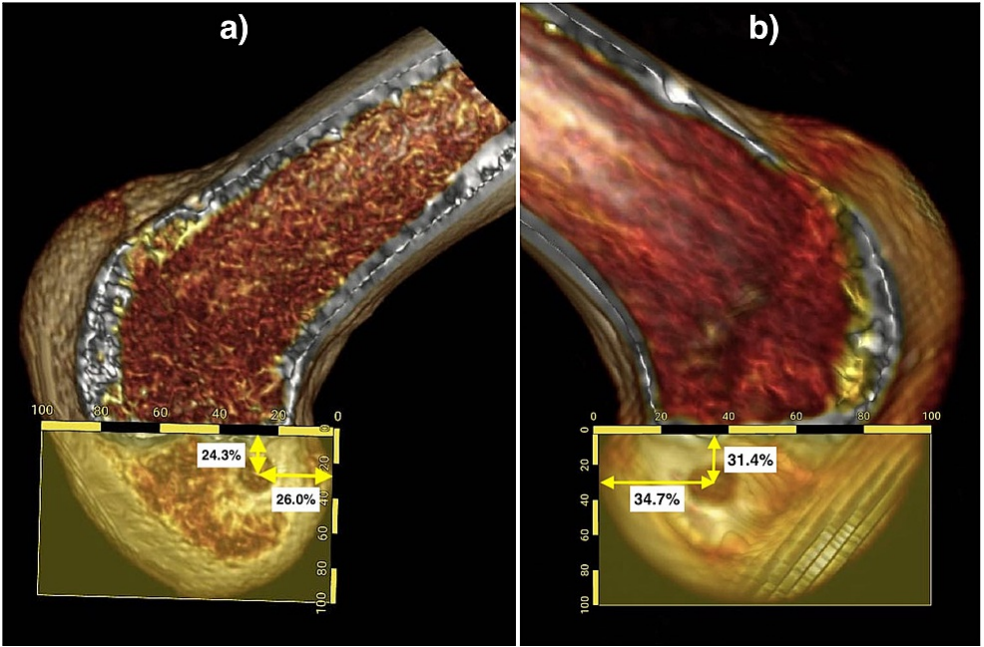
Computed tomography three-dimensional images showing the height and depth of the femoral tunnel entry aperture drilled through the standard anteromedial portal (a), and the far medial portal (b).

Further, we collected the Digital Imaging and Communications in Medicine (DICOM) files of the CT scans of ACLR-operated knee joints. Three-dimensional (3D) reconstruction models of individual knee joints were prepared using OsiriX 10.0.5.1.20 software (Pixmeo SARL, Geneva, Switzerland). The medial condyle was progressively removed from the 3D images to obtain a sagittal view showing both the roof of the intercondylar notch as the Blumensaat line and the medial surface of the lateral condyle of the femur. We used the quadrant method described by Bernard et al. [12] to mark the location of the center of the entry aperture of the femoral tunnel. A rectangle was formed by the Blumensaat's line, a parallel line tangential to the most inferior margin of the lateral condyle, and two perpendicular lines at the shallowest and deepest contour of the lateral femoral condyle along the Blumensaat's line. The femoral tunnel aperture center's depth was measured as its percentage distance from the posterior to the anterior direction in proportion to the overall anterior-posterior extent of the rectangle. The height of the aperture's center was measured as the percentage distance (superior to inferior) from the Blumensaat's line in proportion to the overall superoinferior extent of the rectangle (Figures 1, 2).

The continuous variables were expressed as mean±standard deviation, and categorical variables were expressed as proportions. We created two comparison groups based on the femoral tunnel drilling portal, i.e., standard AM and far medial portals. We compared the demographic parameters and quadrant-based locations of the femoral tunnel aperture between the two groups. The continuous variables were compared using the student t-test, and categorical variables were compared using the Chi-square test. A p-value of less than 0.05 was considered statistically significant.

## Results

Forty-two case records were reviewed. All of them belonged to unilateral ACLR surgery. Twenty-six cases underwent femoral tunnel drilling through the far medial portal and sixteen cases through the standard AM portal. The demographic parameters were comparable, but the tunnels' entry apertures created through the far AM portal were significantly shallower and inferior to the standard AM portal-created femoral tunnels. The detailed results are presented in Table 1.

**Table 1:** A Comparison of demographic variables and femoral tunnel entry aperture among the two arthroscopic portal-based groups. BMI- Body mass index # Data of 26 cases (n =14 for standard anteromedial portal group, and n = 12 for far medial portal group cases) were available ^ Findings of cases with weight and height data that were available for comparison ~ Differences were statistically significant for cases with weight and height data available with p < 0.05.

Variable	All cases (n = 42)	Standard anteromedial portal group (n=16)	Far medial portal group (n=26)	Remarks
Age (in years)	30.3±8.5	32.6±5.7	28.9±9.7	No significant difference
Male: female ratio	34:8	22:4	12:4	No significant difference
Weight (in kg)^#^	66.6±14.2	67.5±12.0	66.1±15.1	No significant difference
Height (in cm)^#^	161.5±5.8	161.4±6.7	161.7±5.4	No significant difference
BMI (in kg/m^2^)^#^	24.0±5.1	24.4±4.5	23.8±5.3	No significant difference
Depth of the femoral tunnel aperture (in %)	27.4±3.0	24.4±1.6 (24.4±1.7)^^^	29.3±1.9 (30.4±2.3)^^^	Statistically significant difference^~^
Height of the femoral tunnel aperture (in %)	32.3±2.9	29.1±1.6 (29.3±1.7)^^^	34.3±1.3 (34.1±1.2)^^^	Statistically significant difference^~^

## Discussion

The evolution of transportal techniques for femoral tunnel drilling is related to the need for independent femoral tunnel drilling for an anatomical ACLR, which had not been possible with the traditional transtibial techniques. A medial arthroscopic portal allows directing the femoral tunnel in a non-vertical location which fulfills the purpose of anteroposterior and rotation stability of the reconstructed ligament. The transportal femoral tunnel drilling through a medial portal is currently the standard method of ACLR [13]. However, several modifications of the medial portal have been described to make femoral tunnel drilling easy and in line with the anatomical ACLR [2,4-9]. The most commonly advocated modification is the further medialization of the medial arthroscopic portal to allow the femoral tunnel to track more orthogonal to the lateral wall of the intercondylar notch [14]. However, medialization adds the hindrance from the medial femoral condyle and the body of the medial meniscus, which can potentially alter the aperture of the femoral tunnel.

Our findings suggest that the femoral tunnels created through the far medial portal were anterior and inferior to those created through the standard AM portal. Based on the quadrant method, there is a difference of approximately 5% in the anteroposterior and superior-inferior positioning of the femoral tunnel entry. The clinical significance of these differences is, however, difficult to predict. In general, there will be excessive graft tension during knee flexion at an anterior entry aperture, resulting in joint stiffness and ACLR failure [10]. Conversely, if the aperture is too deep, there is a risk of posterior wall blowout of the lateral condyle [11]. In addition, when the tunnel is positioned too high, it will lack rotational stability [15], while too low a tunnel has a risk of subchondral bone damage and aperture level blowout.

Current literature is heterogeneous regarding the optimum portal location for femoral tunnel drilling. The medial accessory portal or a far medial portal had been suggested to create a more horizontal tunnel entry which was difficult with the standard AM portal [2,14]. However, there have been concerns that the medial femoral condyle often hinders the drilling tract through the far AM portal, resulting in iatrogenic medial femoral condyle chondral injury [16]. This hindrance can result in missing the desired tunnel track when the reamer is positioned over the guide pin, which can potentially cause a guide pin-reamer track mismatch.

The quadrant method used in our analysis has been a widely used validated method for femoral tunnel aperture assessment [12]. Our findings suggest the standard AM portal results in deeper tunnel aperture while the height is similar to the normal ACL femoral footprint. On the contrary, with the far medial portal, the depth of the tunnel aperture appears to be comparable to the normal ACL footprint. Still, the far medial portal's tunnel entry is inferior, suggesting a horizontal femoral tunnel position. However, there can be wide variations in the original ACL footprint center considering individual and population-based variations in the femoral condylar morphology. These ACL femoral footprint variations can predicted from the findings of Yamamoto et al. [17] ( depth: 27%, height: 29%), Colombet et al. [18] (depth: 23.9%, height: 37.95%), and Guo et al. [19] (depth: 43.1%, height: 38.3%). Furthermore, there is minimal evidence of the influence of portal technique on the femoral tunnel entry aperture location. Our findings of the deeper tunnel with the standard AM portal are similar to those of Erdem et al. [20]. However, the authors could not find any significant differences between the two portals in terms of the superior-inferior location of the tunnel. In our findings, a tendency to keep drill orthogonal to the lateral wall of intercondylar notch could have potentially resulted in inferior positioning of the tunnel. 

There have been some limitations of the current study. First, the study has a limited sample size. The postoperative CT-based assessment of the ACLR has not been routinely performed and vary among surgeons. Therefore, a limited number of CT studies were available for review. Second, the study analyzes the variation of femoral tunnel entry aperture only, using the standard AM and far medial portals. The other tunnel parameters were not analyzed. Other parameters, such as surgeons' technique, local population morphology, especially lateral condylar size, can also affect the tunnel dimensions. Those were not analyzed in this study and would probably need a prospective study. Third, we did not analyze the clinical outcomes. A prospective study with longer follow-up would be desirable for that purpose. Fourth, there can be variations in the positioning of standard AM and far medial portals because of morphological variations of knee and surgeons techniques which can affect the femoral tunnel aperture. However, those variations were beyond the scope of the current study. Nevertheless, the study findings of the significant influence of the femoral tunnel drilling portal on its entry aperture can help surgeons better tailor their femoral tunnel drilling strategy. In addition, further prospective studies addressing the limitations of current retrospective analysis would be required.

## Conclusions

The choice of transportal drilling portals can influence femoral tunnel aperture in ACLR surgery. A tendency towards anterior and inferior positioning of the femoral tunnel entry aperture has been observed when a far medial arthroscopic portal is used for femoral tunnel drilling. Therefore, care must be taken to ensure that the drilling guide pin position does not change when the reamer is passed over it. Further prospective evidence would be required to better characterize the changes in femoral tunnel morphology with the change in the drilling portal and their impact on ACLR outcomes.
